# Diagnosis of the Diseases of Children

**Published:** 1898-02

**Authors:** 


### Diagnosis of the Diseases of Children.

Inspection should be methodical. Posture, character of respiration, appearance of abdomen and particularly the face and the shape of the head demand attention. Suffering is easily detected. The features become pinched and drawn in exhaustive disease. Mouth breathing and nasal discharge are significant. The shape and size of the head, the fontanelles, bridge of nose, pupils, ears, lymph glands of neck, presence of eruptions, herpetic vesicles, etc., must be carefully noted.

Determine whether eruption of the teeth is normal. While no child is ever seriously ill from teething, it often happens that pronounced reflex symptoms develop and continue until the irritation is removed. The child is fretful and peevish, restless at night, and has disturbance of digestive functions. There may be a little rise of temperature. Not infrequently eczema of the cheeks develops or is aggravated by this cause.

Admitting and emphasizing the fact that difficult dentition is one factor in the causation of certain symptoms, I believe the physician can not be too careful or thorough in examination before making a diagnosis of teething.—*Atlantic Medical Weekly.*

